# Decreased expression of ApoF associates with poor prognosis in human hepatocellular carcinoma

**DOI:** 10.1093/gastro/goz011

**Published:** 2019-04-21

**Authors:** Ya-Bin Wang, Bo-Xuan Zhou, Yun-Biao Ling, Zhi-Yong Xiong, Rui-Xi Li, Yue-Si Zhong, Ming-Xing Xu, Yi Lu, Hao Liang, Gui-Hua Chen, Zhi-Cheng Yao, Mei-Hai Deng

**Affiliations:** 1 Department of Liver Transplantation, Third Affiliated Hospital of Sun Yat-sen University, Guangzhou, Guangdong, P. R. China; 2 Department of Hepatobiliary Surgery, Third Affiliated Hospital of Sun Yat-sen University, Guangzhou, Guangdong, P. R. China; 3 Department of General Surgery, The Lingnan Hospital of Sun Yat-sen University, Guangzhou, Guangdong, P. R. China

**Keywords:** hepatocellular carcinoma, biomarkers, apolipoprotein F, prognosis

## Abstract

**Background:**

Hepatocellular carcinoma (HCC) is frequently associated with metabolism dysfunction. Increasing evidence has demonstrated the crucial role of lipid metabolism in HCC progression. The function of apolipoprotein F (ApoF), a lipid transfer inhibitor protein, in HCC is incompletely understood. We aimed to evaluate the functional role of ApoF in HCC in this study.

**Methods:**

We used quantitative reverse-transcription polymerase chain reaction (qRT-PCR) to detect *ApoF* mRNA expression in HCC tissues and hepatoma cell lines (SMMC-7721, HepG2, and Huh7). Immunohistochemistry was performed to detect the expression of ApoF in HCC tissues. The associations between ApoF expression and clinicopathological features as well as HCC prognosis were analyzed. The effect of ApoF on cellular proliferation and growth of SMMC-7721 and Huh7 cells was examined *in vitro* and *in vivo*.

**Results:**

ApoF expression was significantly down-regulated at both mRNA and protein levels in HCC tissues as compared with adjacent tissues. In SMMC-7721 and Huh7 HCC cells, ApoF overexpression inhibited cell proliferation and migration. In a xenograft nude mouse model, ApoF overexpression effectively controlled HCC growth. Kaplan–Meier analysis results showed that the recurrence-free survival rate of HCC patients with low ApoF expression was significantly lower than that of other HCC patients. Low ApoF expression was associated with several clinicopathological features such as liver cirrhosis, Barcelona Clinic Liver Cancer stage and tumor-node-metastasis stage.

**Conclusions:**

ApoF expression was down-regulated in HCC, which was associated with low recurrence-free survival rate. ApoF may serve as a tumor suppressor in HCC and be a potential application for the treatment of this disease.

## Introduction

Hepatocellular carcinoma (HCC) is one of the most common malignant tumors and a leading cause of cancer-related death among patients with liver cirrhosis. Although the treatment of HCC has greatly evolved over time, recurrence after resection or ablation is common owing to intrahepatic metastasis and vascular invasion [1–3]. Recent studies have shown that various oncogenes and tumor suppressor genes contribute to the biological behavior of HCC [4]. Therefore, identification of novel targets and development of effective diagnostic biomarkers are desirable for HCC control.

Apolipoprotein F (ApoF) is one of the minor apolipoproteins found in the blood plasma. ApoF is also known as lipid transfer inhibitor protein, given its ability to inhibit cholesteryl ester transfer protein-mediated transfers of cholesteryl esters and triglycerides [5, 6]. Recent studies have shown that ApoF modulates high-density lipoprotein (HDL) metabolism and that the overexpression of ApoF in mice reduces HDL metabolism by improving the clearance of HDL cholesterol ester from the plasma [7]. Female ApoF-knockout mice tended to have elevated levels of liver cholesteryl esters as compared with the wild-type controls fed with a chow diet [8].

Changes in ApoF expression levels are reported in several diseases. ApoF levels are elevated in hypercholesterolemia and negatively correlated with triglyceride levels. A recent study showed that ApoF levels in HDL were markedly reduced in a small set of subjects with coronary artery disease [9]. Furthermore, ApoF protein levels increased in mice after the combination treatment with statin and niacin [10–12]. Therefore, aberrant ApoF expression may lead to abnormalities in lipid metabolism.

Until now, no study has explored the expression of ApoF in HCC. In the present study, we investigated the expression of ApoF in HCC and explored the association between ApoF level and patient prognosis.

## Materials and methods

### Human-tissue specimens

Samples of fresh HCC tissues and matched adjacent non-tumorous tissues were collected from 116 patients who underwent hepatic resection at the Department of Hepatobiliary Surgery of the Third Affiliated Hospital of Sun Yat-sen University (Guangzhou, China) between January 2010 and December 2012. Tumor specimens obtained from non-necrotic areas of the tumor were snap frozen in liquid nitrogen and stored at −80°C. Ethics agreements for the present study were approved by the Research Ethics Committee of the Third Affiliated Hospital of Sun Yat-sen University and signed informed consent was obtained from each patient. This investigation conforms with the principles outlined in the Declaration of Helsinki.

### RNA extraction and real-time quantitative polymerase chain reaction

Two micrograms of total RNA extracted from tissues using TRIzol reagent (Invitrogen, Carlsbad, California, USA) was reverse transcribed using oligo (dT) primers and reverse transcriptase (Invitrogen, Carlsbad, California, USA). Quantitative reverse-transcription polymerase chain reaction (qRT-PCR) was performed using a SYBR Green Master kit (Takara, Kusatsu, Shiga Ken, Japan) and a LightCycler^®^ 480 system (Roche, Basel, Switzerland). The gene primers were designed by Invitrogen. Relative gene expression was calculated using the formula 2^−ΔCt^ and glyceraldehyde-3-phosphate dehydrogenase (GAPDH) was used as an internal gene for normalization with the following formula: ΔCt (critical threshold) = Ct (ApoF) − Ct (GAPDH). Relative gene-expression levels for HCC tissues and adjacent liver tissues were calculated using 2^−ΔΔCt^.

### Immunohistochemical staining

Paraffin-embedded tissues were first stained with hematoxylin and eosin for histological examination. Sections were subsequently subjected to antigen retrieval by first heating slides in a microwave oven at 100°C in 0.1 mol/L citric acid buffer (pH 6.0), followed by incubation with appropriate antibodies at 4°C overnight. After secondary antibody incubation at 37°C for 1 h, the slides were developed in 0.05% diaminobenzidine containing 0.01% hydrogen peroxidase. As a negative control, specific antibodies were replaced with normal goat serum for co-incubation at 4°C overnight prior to immunohistochemical staining. Staining intensity was independently evaluated by two certified pathologists (Dr Pan and Dr Feng).

The intensity of ApoF expression in tissue samples was evaluated and scored by a certified pathologist. The immunostaining score index was calculated by multiplying cell staining intensity (0, none; 1, weak; 2, moderate; and 3, strong) and the percentage of tissue stained (0–100%). Scores lower than the median score were defined as low expression, whereas those greater than or equal to the median score were classified as high expression in the examined tissues.

### Cell culture and Western blot analysis

The LO2 liver cell line and SMMC-7721, HepG2, and Huh7 HCC cell lines were obtained from the liver disease laboratory of the Third Affiliated Hospital of Sun Yat-sen University (Guangzhou, China). Cells were cultured in Dulbecco’s modified Eagle’s medium (DMEM; Invitrogen, Carlsbad, California, USA) with 10% fetal bovine serum (FBS; Gibco, Carlsbad, California, USA), 100 IU/mL penicillin and 100 µg/mL streptomycin at 37°C in 5% CO_2_ atmosphere. Goat polyclonal anti-ApoF antibody was purchased from Abcam (Cambridge, Massachusetts, USA).

Protein extracts from HCC cells were equally loaded on 10% sodium dodecyl sulfate polyacrylamide gel electrophoresis (SDS-PAGE) gels and electro-transferred onto polyvinylidene fluoride (PVDF) membranes (250 mA for 1.5 h). The membranes were blocked with 5% non-fat milk in Tris-buffered saline containing 0.1% Tween-20 and incubated with indicated primary antibodies. The membranes were treated with appropriate secondary antibodies and the signals were detected using a chemiluminescence Phototope-horseradish peroxidase kit (Pierce Biotechnology, Rockford, Illinois, USA) according to the corresponding manufacturer’s instructions.

### Construction of stable cell lines

The plasmid ApoF-Lv201 and the expression plasmid vector Lv201 were constructed by GeneCopoeia (Guangzhou, Guangdong, China). Lipofectamine 2000 (Invitrogen, Carlsbad, California, USA) was used to transfect ApoF-Lv201 and Lv201 vectors into SMMC-7721 and Huh7 cells. After 48 h from transfection, the stable transfectants were selected by using 800 µg/mL of G418 (Mpbio, Santa Ana, California, USA); these cells were named as SMMC-7721-ApoF, Huh7-ApoF, SMMC-7721-vector, and Huh7-vector.

### Cell proliferation and transwell assays

Control cells and cells stably expressing ApoF were plated into 96-well plates at a density of 1000 cells/well in DMEM with 10% FBS. At indicated time points, cell proliferation was determined using a cell-counting kit-8 (CCK-8; DOJINDO, Kumamoto, Japan) assay.

Transwell assays were conducted using Millicell hanging BioCoat Matrigel and control chambers from BD Bioscience (24-well insert, 80-µm pore size). Briefly, 25 × 10^4^ cells in 200 µL of serum-free DMEM were loaded into the upper chambers of the transwell device. A total of 0.5 mL DMEM supplemented with 10% FBS was loaded into the lower chamber. After 24 h of treatment, the cells on the underside of the membrane were stained and counted under a microscope in five random high-power fields.

### Xenograft nude mice

Control cells (MHCC97), also provided by the liver disease laboratory of the Third Affiliated Hospital of Sun Yat-sen University, Guangzhou, China, and ApoF-stably-expressing cells (MHCC97-ApoF) were adjusted to a density of 5 × 10^6^ cells in 100 µL phosphate-buffered saline. These cells were subcutaneously injected into the flank of nude mice (6 weeks old, male, *n *=* *5; Sun Yat-sen University Laboratory Animal Center, Guangzhou, China) to develop xenograft mouse models. All mice were sacrificed at the end of 6 weeks after injection. For tumor-growth assay, tumor length and width were measured using a caliper every week, and tumors were completely isolated from the mice and then weighed using an electronic scale. Tumor volume was calculated as follows: tumor volume (mL) = width^2^ × length × 0.5. All animal studies were performed in accordance with ARRIVE (Animal Research: Reporting In Vivo Experiments) guidelines of the UK and were approved by the Institute Research Medical Ethics Committee of Sun Yat-sen University.

### Statistical analysis

Statistical analysis was performed using SPSS 22.0 (Chicago, Illinois, USA). The data in different groups were compared using χ^2^ tests, two-tailed Student’s *t*-test, Kaplan–Meier plots, and Fisher’s exact test. Cumulative survival time was calculated using the Kaplan–Meier method and analyzed using log-rank tests. Data are presented as means ± standard error of the mean. The threshold used for statistical significance was *P *<* *0.05.

## Results

### *ApoF* expression is down-regulated in HCC samples

**Figure 1.**
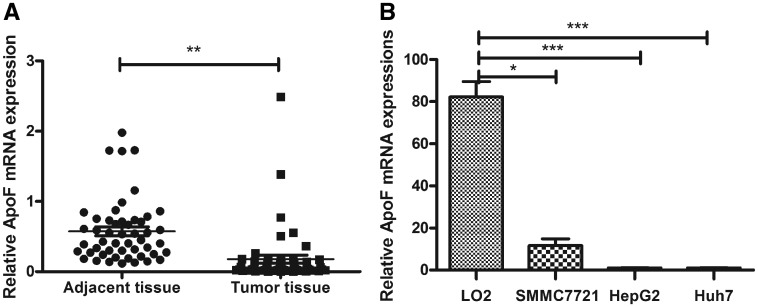
*ApoF* expression is decreased in HCC tissues and cell lines. (**A**) *ApoF* mRNA expression in 50 pairs of tumor and tumor-adjacent tissue samples, as determined using real-time PCR. *GAPDH* was used as a loading control (*P <* *0.05, **P <* *0.01, ***P <* *0.001). (**B**) *ApoF* mRNA expression in LO2, SMMC-7721, HepG2, and Huh7 cells.

To investigate the role of ApoF in HCC, we examined *ApoF* mRNA expression in 50 randomly selected pairs of HCC-tissue and adjacent liver-tissue samples. We found that *ApoF* expression was significantly down-regulated in the tumor tissues when compared with the expression level in the adjacent non-tumor tissues (Figure 1A).

**Figure 2.**
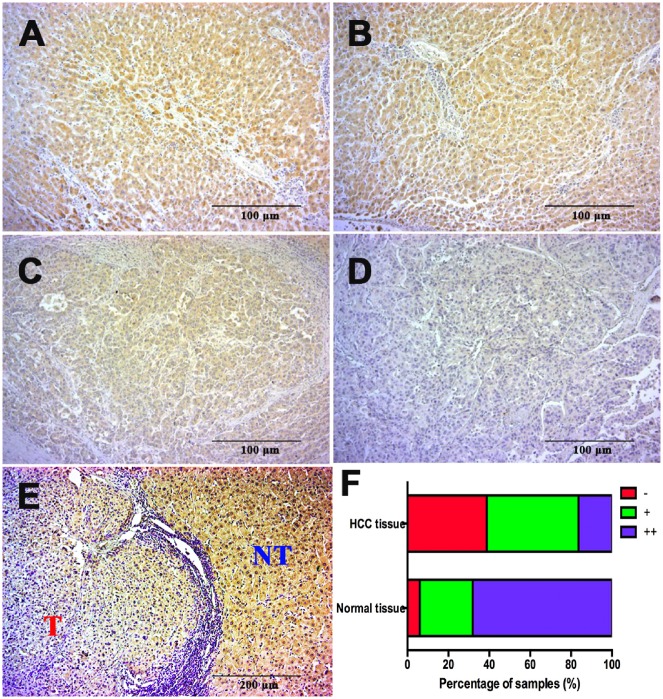
ApoF protein expression is abnormally reduced in HCC tissues (magnification, ×100). (**A**) and (**B**) Representative photographs of strongly positive (++) staining for ApoF protein in normal liver tissue. (**C**) and (**D**) Representative photographs of weakly positive (+, (**C**)) and negative (−, (**D**)) staining for ApoF protein in HCC tissue. (**E**) Representative contrast between a tumorous area (‘T’) and an adjacent non-tumorous area (‘NT’). (**F**) Distributions of ApoF staining grades (−, +, and ++) in normal liver tissue and HCC tissue. Bars* *=* *100 μm.

To confirm this result with more samples, we examined ApoF expression by immunohistochemistry in 116 pairs of HCC-tissue and adjacent liver-tissue samples. Results of immunohistochemistry demonstrated the localization of ApoF in the cytoplasm. Of 116 HCC samples, 18 were strongly positive for ApoF expression; 84.5% of HCC samples exhibited weak staining or negative staining (Figure 2); on the contrary, ApoF antibody staining results were positive in almost 95% of adjacent liver tissues.

### ApoF expression in HCC cell lines affects the *in vitro* growth and migration of cells

As ApoF expression was down-regulated in HCC tissues, we investigated whether ApoF expression affects cell growth and migration. We determined the mRNA and protein expression of ApoF in HCC cell lines (SMMC-7721, HepG2, and Huh7) and normal liver cell line (LO2) and found that HepG2 and Huh7 cells exhibited low ApoF expression, whereas SMMC-7721 cells exhibited moderate ApoF expression; LO2 cell line showed high ApoF expression (Figure 1B).

**Figure 3.**
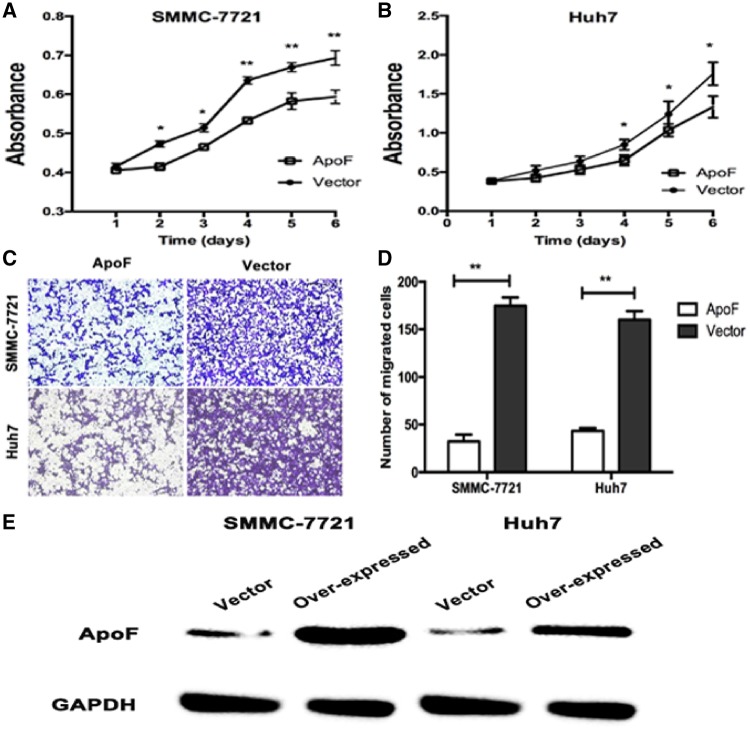
ApoF overexpression affects cellular proliferation and migration *In vitro*. (**A**) and (**B**) Representative images showing the inhibition of cell proliferation *in vitro* after ApoF overexpression, as analysed with CCK-8 assay. (**C**) and (**D**) Transwell migration of SMMC-7721 and Huh7 cell lines stably transfected with ApoF or empty vector (with cells exhibiting migration indicated in the histogram). (**E**) Western blot analysis to detect ApoF expression in stable cell lines (SMMC-7721-ApoF and SMMC-7721-vector; Huh7-ApoF and Huh7-vector). GAPDH was used as a loading control.

Therefore, we determined the effects of ApoF expression on the growth of SMMC-7721 and Huh7 cells *in vitro*. The results of CCK-8 proliferation assay showed that the proliferation rates of SMMC-7721-vector and Huh7-vector cells were higher than those of SMMC-7721-ApoF and Huh7-ApoF, respectively (*P *<* *0.05; Figure 3A and B).

To investigate the effects of ApoF expression on the migration of HCC cells, we performed transwell migration assays. The migration of SMMC-7721-ApoF and Huh7-ApoF cells was slow through the Matrigel-coated inserts following ApoF expression up-regulation (Figure 3C and D).

The expression of ApoF protein was detected with anti-ApoF and anti-GAPDH antibodies at Day 6. Western blot results showed that ApoF protein expressions in SMMC-7721-vector and Huh7-vector cells were down-regulated compared with those in SMMC-7721-ApoF and Huh7-ApoF cells (Figure 3E). Taken together, these results suggest that *ApoF* may perform the function of a tumor suppressor.

### Decreased ApoF expression predicts poor prognosis in patients with HCC

**Figure 4.**
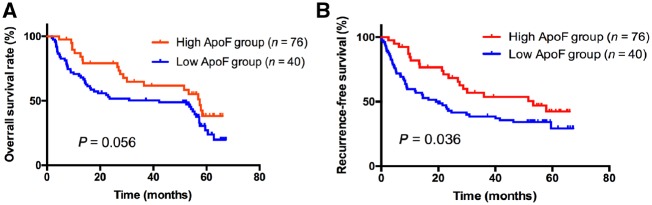
Kaplan–Meier survival analysis according to ApoF protein expression in patients with HCC. (**A**) Overall survival rate and (**B**) recurrence-free survival rate. Recurrence-free survival rate was significantly lower in the low-ApoF expression group (solid line) than in the high-ApoF expression group (dotted line) (*P *=* *0.036).

To explore the association of clinicopathological factors with ApoF down-regulation in HCC, we performed immunohistochemistry. As shown in Figure 4, patients were divided into low and high expression groups based on their ApoF protein expression levels. As shown in Table 1, low ApoF expression was significantly associated with liver cirrhosis (*P *=* *0.007), Barcelona Clinic Liver Cancer (BCLC) stage (*P *=* *0.020) and tumor-node-metastasis (TNM) stage (*P *=* *0.049). However, ApoF expression showed no significant association with age, gender, serum AFP (α-fetoprotein) level, vascular invasion, tumor number, tumor differentiation or tumor size (Table 1).

**Table 1.** Associations between ApoF expression and clinicopathological characteristics in 116 patients with HCC

Variable	No. of cases	ApoF expression		χ^2^ value	*P*-value
		Low (*n* = 40)	High (*n* = 76)		
Age (years)					
≤50	62	18	44	1.751	0.186
>50	54	22	32		
Sex					
Male	98	32	66	0.936	0.333
Female	18	8	10		
HBsAg					
Positive	71	24	47	0.037	0.847
Negative	45	16	29		
AFP (ng/mL)					
≤400	69	28	41	2.802	0.094
>400	47	12	35		
Liver cirrhosis					
Absent	36	6	30	7.3334	0.007
Present	80	34	46		
Vascular invasion					
Absent	88	31	57	0.089	0.765
Present	28	9	19		
Tumor size					
≤5 cm	82	29	53	0.097	0.756
>5 cm	34	11	23		
Tumor number					
Single	98	35	63	0.424	0.515
Multiple	18	5	13		
Tumor differentiation					
Well	33	13	20	0.492	0.483
Moderate–poor	83	27	56		
BCLC stage					
0/A	61	27	34	5.446	0.020
B/C	55	13	42		
TNM stage					
I/II	76	31	45	3.880	0.049
III/IV	40	9	31		

AFP, alpha fetoprotein; BCLC, Barcelona Clinic Liver Cancer; TNM, tumor-node-metastasis; HBsAg, hepatitis B surface antigen.

We analyzed the relationship between survival time and ApoF expression. Lower ApoF expression was significantly associated with worse outcome. Besides, the patients with low ApoF expression tended to have a short recurrence time.

### Overexpression of ApoF controls the tumor growth *in vivo*

**Figure 5.**
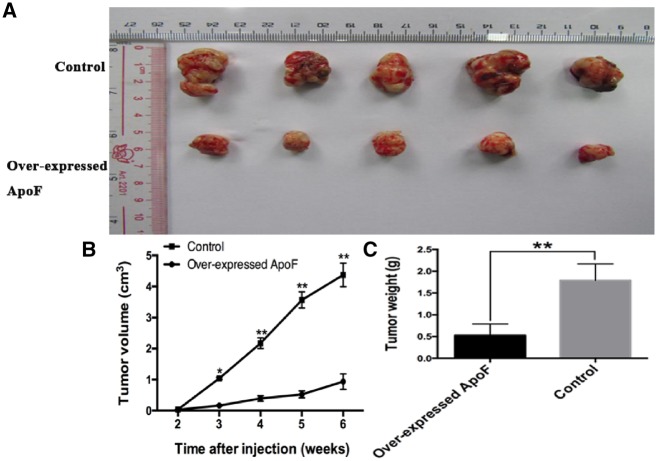
Overexpression of ApoF-controlled tumor growth. (**A**) Tumors were harvested at 6* *weeks and the tumor size was reported. (**B**) Tumor volume was calculated every week after injection. (**C**) Tumor weight was calculated after sacrificing the mice. The data indicate that the xenograft tumors grew larger and faster in the control group than in the ApoF-overexpressing group.

As shown in Figure 5, MHCC97 cells overexpressing ApoF were subcutaneously injected into the flank side of the right leg. The growth of tumors was significantly inhibited in mice carrying ApoF-overexpressing xenografts as compared with those from the control group (Figure 5A). Tumor growth, evaluated with tumor volume and weight, was significantly decreased in mice carrying ApoF-overexpressing xenograft as compared with those from the control group (*P *<* *0.001) (Figure 5B and C).

## Discussion

In this study, we demonstrated that ApoF expression was significantly decreased in HCC tissues and cell lines and that ApoF localizes in the cell cytoplasm. Furthermore, low ApoF expression was highly associated with several clinicopathological indicators such as liver cirrhosis, BCLC stage, and TNM stage. Kaplan–Meier curves showed that the patients with low ApoF expression had shorter recurrence-free survival than other patients.

To investigate the function of ApoF in the progression of HCC, we constructed two stably transfected HCC cell lines that over-expressed ApoF. Our results revealed that ApoF expression inhibited the proliferation of SMMC-7721 and Huh7 cells *in vitro*. In transwell migration assay, SMMC-7721 and Huh7 cells migrated slowly after ApoF expression up-regulation. We also proved that the overexpression of ApoF significantly inhibited the tumor growth *in vivo*.

ApoF-positive cells were defined as liver cells with cytoplasmic ApoF expression. The adjacent non-tumorous tissues exhibited higher expression of ApoF. As many patients had liver cirrhosis, most samples exhibited heterogeneity with respect to positive staining for ApoF expression. In most HCC tissues, the rate of immunohistochemical positivity for ApoF expression was rather low.

The liver plays a key role in the metabolism of plasma apolipoproteins, and plasma lipid profiles may be altered in HCC [13, 14]. In most reports on HCC, apolipoprotein E showed a slight to significant increase in patients with HCC [15, 16]. Plasma levels of apolipoproteins may serve as a sensitive marker of hepatic impairment [17].

The members of the C/EBP family are important players involved in the regulation of ApoF expression in hepatoma cell lines [18]. The C/EBP family comprises six related proteins from the larger family of basic region leucine zipper (bZIP) transcription factors; among these proteins, C/EBPα and C/EBPβ expression is enriched in the liver [19–21]. C/EBPα may increase ApoF promoter activity in Huh7 cells, whereas C/EBPβ overexpression had no effect on the activity of ApoF promoter in HepG2 and Huh7 cells. C/EBPα alone is thought to specifically activate the ApoF promoter. We speculate that the mutation of the C/EBP-binding site may almost completely abolish ApoF promoter activity in HCC.

This study has a few limitations. First, this investigation included a relatively small number of cases; although adequate statistical significance was achieved in this study, further studies with larger numbers of patients would be beneficial. Second, although high ApoF expression was significantly associated with cell proliferation and migration *in vitro* and *in vivo*, the specific biological mechanisms underlying this association are unclear. Animal studies are necessary to confirm the role of ApoF in HCC cells. Third, this study did not reveal the role of ApoF in metabolism or cholesterol regulation; further research is warranted to evaluate this function of ApoF.

In conclusion, we identified a cancer-related gene and explored the function of ApoF in tumor biology. Low ApoF expression was an independent predictor of recurrence for patients with HCC. ApoF overexpression may reduce HCC growth and migration *in vitro* and *in vivo*. Next, we plan to investigate the detailed mechanisms underlying the effects of ApoF on cell proliferation and migration. Furthermore, the relationship between lipid metabolism and ApoF in HCC will be explored in future studies, which may provide a new approach for the therapy of HCC from the perspective of energy metabolism.

## Authors’ contributions

Y.B.W., B.X.Z., and Y.B.L. contributed equally as first authors. Z.C.Y., Y.B.L., and M.H.D. conceived and designed the project. Y.B.W., B.X.Z., and Z.C.Y. acquired the data, while Z.Y.X., R.X.L., Y.S.Z., M.X.X., Y.L., H.L., and G.H.C. analyzed and interpreted the data; B.X.Z., and Z.C.Y. drafted the manuscript. All authors reviewed and approved the final manuscript.

## Funding

This study was supported by grants from the National Natural Science Foundation of China [No. 81572726], the Natural Science Foundation of Guangdong Province [No. 2018A030313641 and No. 2016A030313848], the Science and Technology Planning Project of Guangdong Province [No. 2014A020212122 and No. 2016A020212004] and the Medical Research Foundation of Guangdong Province [No. A2016312].
